# Skin metastasis of malignant mesothelioma^☆^^☆☆^

**DOI:** 10.1016/j.abd.2020.07.020

**Published:** 2021-07-23

**Authors:** Tatsuhiko Mori, Toshiyuki Yamamoto

**Affiliations:** Department of Dermatology, Fukushima Medical University, Fukushima, Japan

Dear Editor,

Cutaneous metastasis of malignant pleural mesothelioma is rare. We herein report a case of malignant mesothelioma with distant cutaneous metastasis.

A 78-year-old man, who was working as a welder and was exposed to asbestos, visited the Internal Medicine department at our hospital while complaining of shortness of breath for the previous six months. He underwent an aspiration biopsy of the right chest wall. Neoplastic monomorphous cells with eosinophilic or vacuolated cytoplasm and atypical nuclei arranged in a solid or cobblestone pattern. Immunohistochemistry was strongly positive for epithelial membrane antigen and vimentin, focal positivity for AE1/3, calretinin, D2-40, and Wilms tumor-1, but negative for carcinoembryonic antigen, thyroid transcription factor-1, and cytokeratin 5/6. A diagnosis of malignant pleural mesothelioma was made (T3N0M0, stage 3), and the patient was treated with chemotherapy (pemetrexed and carboplatin). However, five months after finishing chemotherapy, he noticed a firm nodule on the left axilla and was referred to our department. Physical examination showed a solitary dermal nodule on the left axilla. Histological examination of the totally resected tumor showed similar atypical epithelioid tumor cells with a number of mitoses (Fig. 1). Immunohistochemistry showed a similar pattern with pleural biopsy with intense staining for vimentin (Fig. 2), as well as focal positivity for AE1/AE3 and D2-40; however, calretinin, a marker of mesothelioma cell, was not detected. Thereafter, three months later, skin nodules further appeared on the lower abdomen (two nodules sized 12 mm and 17 mm in diameter) (Fig. 3a), on the left chest (sized 20 mm one nodule) (Fig. 3b), and on the right chest (50 × 35 mm one nodule). Chemotherapy was ceased and best supportive care was chosen.

**Figure 1:**
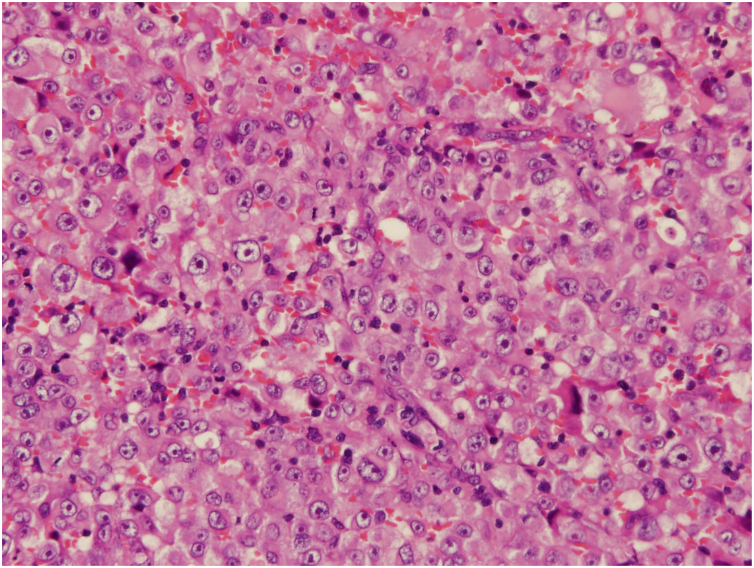
Histological features show neoplastic monomorphous atypical cells, with eosinophilic or vacuolated cytoplasm proliferation in a solid pattern (Hematoxylin & eosin, ×400).

**Figure 2:**
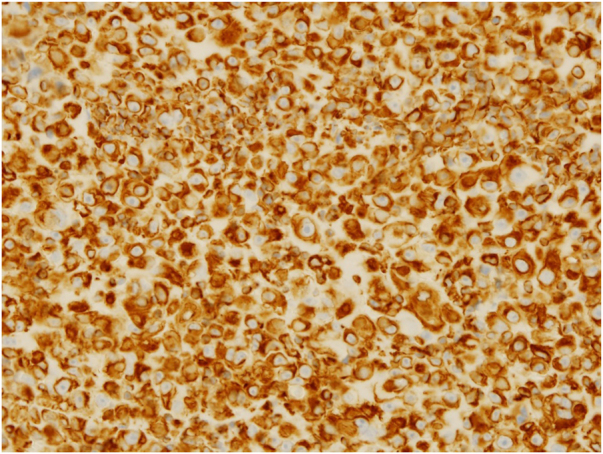
Strong immunopositivity for vimentin (Vimentin, ×400).

**Figure 3:**
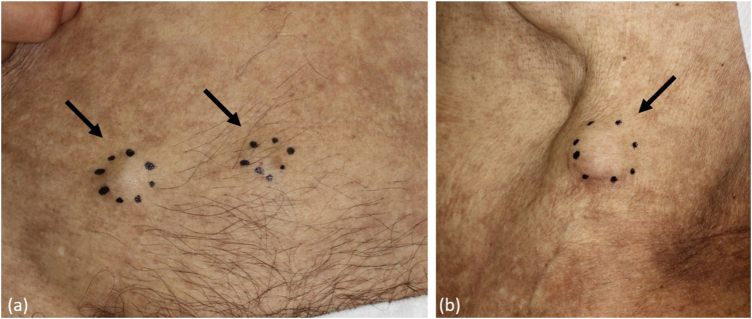
Clinical appearance.

Malignant mesothelioma is a rare neoplasm of the serosal membranes predominantly of the pleura and peritoneum. The incidence of malignant mesothelioma is increasing, especially in patients exposed to asbestos. The current patient had been working in construction areas for a long time, which therefore led to his exposure to asbestos.

Cutaneous metastasis of malignant mesothelioma is relatively rare and can occur in three different ways; i) Regional spread via lymphatics, ii) Direct extension within surgical scars such as needle track, and iii) Distant metastasis via hematogenous spread.^1^ In the present case, a metastatic nodule appeared on the axilla, irrelevant to the aspiration biopsy site. Distant skin metastasis is relatively rare. Ward et al.^1^ reviewed 20 cases of malignant mesothelioma metastatic to the skin, excluding cases of direct extension or regional spread. The average time from the original diagnosis of malignant mesothelioma to cutaneous metastasis was six months. The most commonly involved site was the face, followed by the scalp and chest. Metastatic skin lesions mostly occur as subcutaneous nodules, and multiple lesions are often observed.^1^ In the present case, subcutaneous nodules increased in number within only three months. Skin lesions can also be disseminated.^2^ In the present case, skin metastasis initially appeared five months after the diagnosis of malignant pleural mesothelioma; however, metastasis can be delayed for as long as four years.^3^ By contrast, skin metastasis can occur as an initial manifestation.^2^, ^4^

It was reported that the positive percentage of calretinin expression in malignant pleural mesothelioma was various (50%–100%), depending on the type of calretinin antibody.^5^ The diagnosis of malignant mesothelioma is often challenging, especially in cases of calretinin negative staining. In the present case, the patient’s working history of asbestosis exposure and differentiation from pulmonary adenocarcinoma led to the diagnosis of malignant mesothelioma.

## Financial support

None declared.

## Authors’ contributions

Tatsuhiko Mori: Designed the study; performed the research and contributed to analysis and interpretation of data; wrote the initial draft of the manuscript; approved the final version of the manuscript.

Toshiyuki Yamamoto: Designed the study; assisted in the preparation of the manuscript; approved the final version of the manuscript.

## Conflicts of interest

None declared.
